# Trends and determinants of minimum dietary diversity among children aged 6–23 months: a pooled analysis of Indonesia Demographic and Health Surveys from 2007 to 2017

**DOI:** 10.1017/S1368980021004559

**Published:** 2022-07

**Authors:** Bunga A Paramashanti, Tanvir M Huda, Ashraful Alam, Michael J Dibley

**Affiliations:** 1 Sydney School of Public Health, Faculty of Medicine and Health, The University of Sydney, NSW 2006, Australia; 2 Department of Nutrition, Faculty of Health Sciences, Universitas Alma Ata, Yogyakarta 55183, Indonesia

**Keywords:** Minimum dietary diversity, Food group, Infant and young child feeding, Trends, Indonesia

## Abstract

**Objective:**

To examine minimum dietary diversity (MDD) trends and determinants among children aged 6–23 months.

**Design:**

Secondary analysis of the Indonesia Demographic and Health Surveys (IDHS) between 2007 and 2017. The primary outcome was MDD, the consumption of at least five out of eight food groups (MDD-8). We included a total of 5015 (IDHS 2007), 5050 (IDHS 2007) and 4925 (IDHS 2017) children aged 6 to 23 months to estimate trends of MDD-8 and to identify factors associated with MDD-8. We used multiple logistic regression analysis adjusted for the complex sampling design to investigate the association between the study factors and MDD-8.

**Setting:**

Indonesia.

**Participant:**

A total of 14 990 children aged 6–23 months.

**Results:**

Over the 10 years, the percentage of children who consumed a diversified diet was 53·1 % in 2007, 51·7 % in 2012 and 53·7 % in 2017. Multivariate analyses showed that older age children, higher maternal education, maternal weekly access to media, paternal non-agricultural occupation, history of at least four antenatal care visits and wealthier households were associated with the increased odds of MDD-8. Children living in rural areas, Sulawesi and Eastern Indonesia, were less likely to eat a diversified diet.

**Conclusions:**

The proportion of children meeting MDD-8 has stagnated in the last decade. Child, parental, health care, household and community factors are associated with MDD-8. Therefore, nutrition education programmes and behaviour change communication activities should target mothers and families from socio-economically and geographically disadvantaged populations.

Child undernutrition remains a public health nutrition issue globally and in Indonesia. Approximately 144 million or 21·3 % of children under 5 years of age worldwide experienced stunting in 2019, while wasting continued to affect an estimated 47 million (6·9 %) children^(1)^. In Indonesia, the most recent survey conducted by the Ministry of Health reported the prevalence of stunting was 30·8 % in 2018^(2)^, which was ‘very high’^(3)^. This prevalence has decreased slightly from the previous static level of around 37·0 % between 2007 and 2013^(4)^. The WHO global target is to reduce stunting by 40·0 % in 2025, or a 3·9 % relative reduction per year since 2012^(5)^, but the national rate of decline in Indonesia of approximately 1·3 % per year between 2013^(4)^ and 2018^(2)^ will not be sufficient to reach stunting reduction goal. In addition, the small drop in wasting, from 12·1 % in 2013^(4)^ to 10·2 % in 2018^(2)^, was far from the global wasting reduction target of less than 5·0 % in 2025^(5)^.

A diversified diet may help children fulfil their nutrient demands for optimal nutritional status^(6)^. Overall, dietary diversity mirrors dietary quality and quantity and food security^(7)^ and is a helpful indicator of the level of micronutrient adequacy^(8)^. It is one of the recommended measures for the Global Nutrition Monitoring Framework, which is essential to monitor global nutrition targets^(9)^. In 2008, the WHO advised that all infants and young children should have received at least four of seven food groups (minimum dietary diversity (MDD-7)): (1) grains, roots and tubers; (2) legumes and nuts; (3) dairy products; (4) flesh foods; (5) eggs; (6) vitamin A-rich fruits and vegetables; and (7) other fruits and vegetables^(10)^. In 2017, the WHO Technical Expert Advisory group on nutrition Monitoring (TEAM) revised its definition to include the breastmilk component. Thus, MDD is now a child receiving a minimum of five of eight food groups (MDD-8)^(9)^.

The percentage of children meeting the MDD-8 in low- and middle-income countries was low, ranging from 18 % in the sub-Saharan African region to 54 % in the Latin America and Caribbean region. In the South and Southeast Asia regions, only 29 % of children met the recommended MDD-8^(11)^. The shift from MDD-7 to MDD-8 might lower the percentage of children meeting the dietary diversity, particularly in countries with suboptimal breast-feeding coverage at the age of 2 years^(12)^. In Indonesia, the percentage of children with MDD-7 has stagnated between 2012 (58 %)^(13)^ and 2017 (60 %)^(14)^. Similarly, continued breast-feeding at the age of 2 years has remained steady at 55 % during this period^(13,14)^. No study has estimated the trends with the new MDD-8 in Indonesia.

A secondary analysis using the 2017 Indonesia Demographic and Health Survey (IDHS) found that MDD-7 was associated with the children’s age, maternal education, maternal access to information and household wealth^(15)^. A pooled analysis of the 2012 and 2017 IDHS showed that the increased dietary diversity score was related to the children’s age, antenatal care (ANC) visits, maternal workforce participation, maternal knowledge, region and household wealth^(16)^. In line with previous studies in Indonesia^(16)^, Cambodia^(17)^, Myanmar^(17)^ and Ethiopia^(18)^, household wealth was the only variable that was consistently linked to MDD-7, whereas other factors showed inconsistent results. To our knowledge, no Indonesian research has examined factors associated with MDD-8.

Understanding the trends and determinants of dietary diversity may help explain the progress towards global infant and young child nutrition targets, thus improving the design of programme interventions^(19)^. Moreover, it is critical to conduct a study using the MDD-8 indicator due to the country’s transition to the new measure and the need for a valid interpretation of dietary diversity trends and determinants^(12)^. Thus, this study aimed to examine the trends and determinants of MDD-8 among children aged 6–23 months using nationally representative data collected from the 2007, 2012 and 2017 IDHS. Given the broad application of the previous dietary diversity measure, we also analysed the determinants of MDD-7 to enable comparisons across studies and programmes.

## Methods

### Data sources and study population

We used data from the IDHS collected in 2007, 2012 and 2017^(13,14,20)^. Administratively, Indonesia consisted of thirty-three provinces in 2007 and 2012 and thirty-four provinces in 2017. Below, the province level were districts, subdistricts and villages classified as rural or urban areas.

The total sample size was 14 990 consisted of 5015 children from the IDHS 2007, 5050 children from the IDHS 2012 and 4925 children from the IDHS 2017. Samples were selected using multistage stratified sampling. In the first stage, the survey used probability proportional to the number of households to choose subdistricts. The census blocks or sample clusters were selected from each subdistrict using systematic random sampling in the second stage. The survey team identified twenty-five households in each cluster by simple random sampling and interviewed all ever-married women in the selected households^(13,14,20)^. We restricted our analyses to ever-married women aged 15–49 years whose last-born child was alive and aged 6–23 months.

### Outcome variables

The primary outcome was MDD among children aged 6–23 months throughout the previous day. Based on the WHO-TEAM in 2017, we defined MDD as the consumption of food from at least five of eight food groups (MDD-8): (1) grains, roots and tubers; (2) legumes and nuts; (3) dairy products; (4) flesh foods; (5) eggs; (6) vitamin A-rich fruits and vegetables; (7) other fruits and vegetables; and (8) breastmilk^(9)^. The current indicator was an updated version of MDD-7 in 2008^(10)^ that defined the consumption of at least four of seven food groups without adding breastmilk. For this study analysis of MDD-8, we constructed the breastmilk item based on the separated question of current breast-feeding status. Table 1 presents detailed food group components.

**Table 1:** Dietary diversity food group components

No.	Food groups	Dietary diversity components	
		MDD-7*	MDD-8†
1.	Grains, roots and tubers	✓	✓
2.	Legumes and nuts	✓	✓
3.	Dairy products	✓	✓
4.	Flesh foods	✓	✓
5.	Eggs	✓	✓
6.	Vitamin A-rich fruits and vegetables	✓	✓
7.	Other fruits and vegetables	✓	✓
8.	Breastmilk	–	✓

MDD, minimum dietary diversity.

* Based on the WHO indicator of MDD of at least four of seven food groups in 2008^(10)^.

† Based on the WHO indicator of MDD of at least five of eight food groups in 2017^(9)^.

**Fig. 1:**
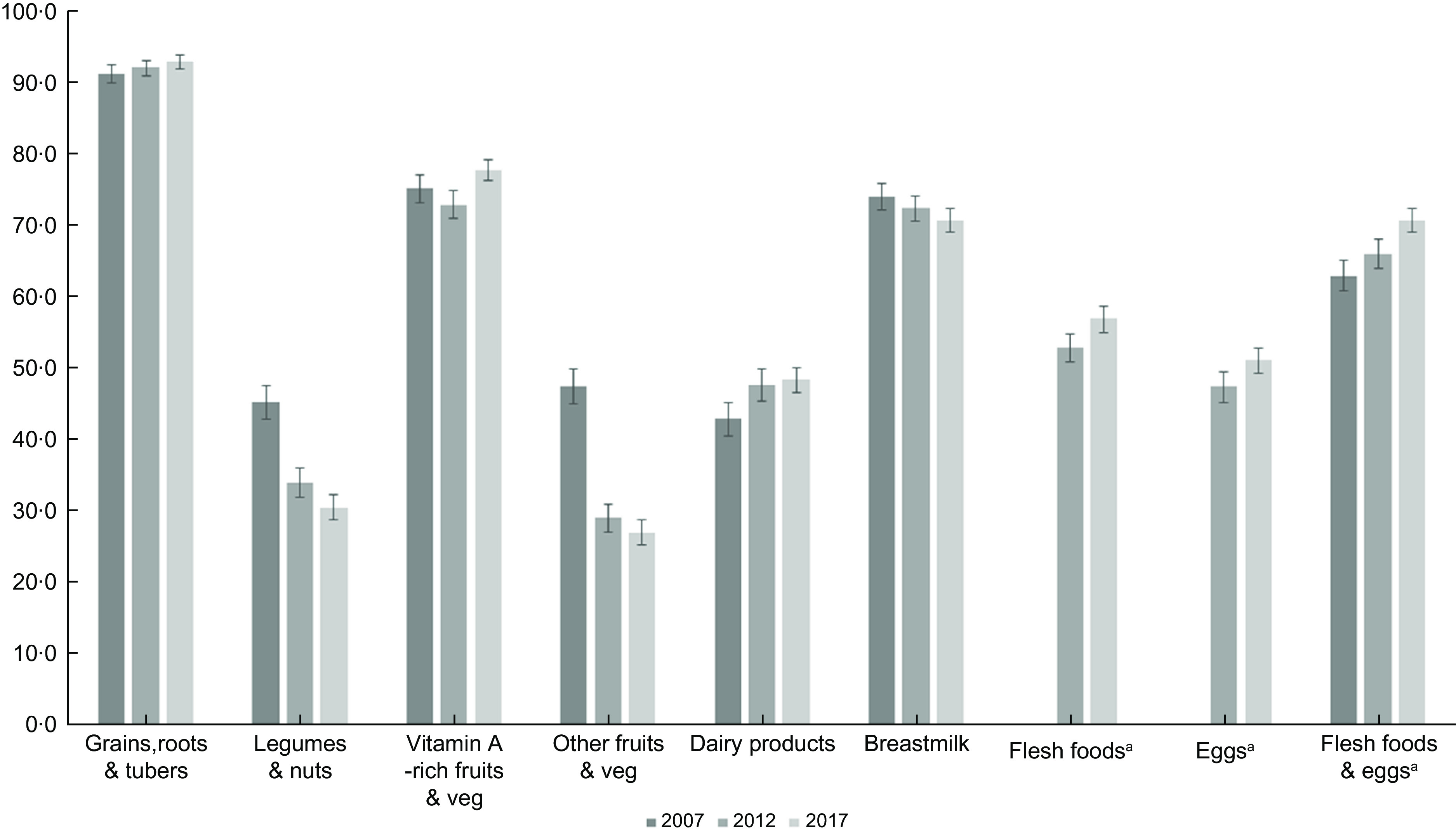
Trends of each food group consumption among children aged 6–23 months

Since the 2007 IDHS collected information on flesh foods and eggs into one single question, we could not assess flesh foods and eggs trend separately in this survey. Thus, we included the combined flesh foods and eggs in Fig. 1 to compare food group intake patterns over the survey year. We found a similar trend for each ‘flesh foods’ and ‘eggs’ and ‘combined flesh foods and eggs’ in the 2012 and 2017 surveys, which were all increasing. We also checked the determinants of MDD-8 for the 2012 and 2017 surveys, either separating or combining the flesh foods and eggs. Since the results were similar, we kept the combined flesh foods and eggs in the 2007 IDHS but separated flesh foods and eggs for the 2012 and 2017 IDHS to analyse factors associated with MDD-8.

### Independent variables

We identified factors potentially related to dietary diversity based on earlier studies in Indonesia^(16)^ and Southeast Asia^(17)^ and the availability of variables across the three surveys^(13,14,20)^. We grouped independent variables as child, maternal, paternal, and health care, household and community factors. Child factors were age (6–11 months, 12–17 months and 18–23 months) and sex (male and female). Maternal factors included age (< 25 years, 25–34 years and ≥ 35 years), education, occupation, media access and decision-making involvement. Paternal factors included education and employment. We categorised the mother’s and father’s education as none or incompleted primary school, completed primary school, completed secondary school and completed higher education. We classified the mother’s occupation into three categories: ‘working in the agricultural sector’, ‘working in the non-agricultural sector’ and ‘not working’. Since there were few unemployed fathers, we developed two categories: ‘not working or working in the agricultural sector’ and ‘working in a non-agricultural sector’. Maternal access to media indicated the mother’s exposure to mass media, such as reading newspapers/magazines, listening to the radio or watching television at least once a week. We constructed maternal involvement in the decision-making based on the final say on one of the following situations: accessing health care for themselves, making major household purchases, or visiting their family and relatives. We considered mothers participated in the decision if they decided by themselves or jointly with their husbands. Health care, household and community factors covered the frequency of ANC visits (< 4 and ≥ 4), the number of children under 5 years of age in the household (≤ 2 and > 2), household wealth, living residency (urban and rural) and region. The DHS used the wealth index as a proxy indicator of household socio-economic status. However, the original index was calculated for the national population, which might cause an urban bias. For this issue, we developed a composite household wealth index using the principal component analysis to weight the specific items from a list of household assets and facilities adjusted for the urban and rural populations^(21)^. We estimated the household wealth index as the sum of the weighted scores, then categorised it into five quintiles: poorest, poorer, middle, richer and richest. We grouped provinces into five regions based on their geographical location: Java and Bali, Sumatera, Kalimantan, Sulawesi, and Eastern Indonesia.

### Statistical analysis

We used Stata version 16.1 (StataCorp) for all statistical testing and applied ‘svy’ commands to adjust the complex sampling design. To set up the survey design, stratification and weighting, we created an identifier for each survey before combining them into a single file. We used these unique identifiers for strata (a combination of provinces and rural–urban residency) and clusters (primary sampling units). We also divided the sampling weight by 1 000 000, since the weight variable in the DHS datasets does not have decimal points^(22)^.

The ‘svy’ command used Taylor series linear approximation to estimate the 95 % CI around the proportions of dietary diversity and each food group^(23)^. We used univariate logistic regression to examine each determinant’s effect on dietary diversity measured by the unadjusted OR. Variables with the significance level of *P* < 0·25 were entered into the multiple logistic regression to create the full baseline model. We set the *P*-value cut-off higher to allow the inclusion of more potentially critical variables in the model. However, we set the survey year, child’s sex and parental covariates, such as mother’s age^(24–26)^, mother’s occupation^(24,26,27)^, mother’s decision-making^(25,27)^, mother’s access to media^(26,28)^ and father’s education^(24,26)^ as fixed variables in the multivariate analysis regardless of their significance. We removed the least important variables one by one using a manual backward elimination beginning with the full model. We also checked for possible interaction for variables: survey year, household economic status, mother’s education, access to media and residency in the final model and removed non-significant interactions. The significance level was set at *P* < 0·01 for the interactions. We reported the adjusted OR with 95 % CI in the final model.

## Results

### Trends of minimum dietary diversity among children and their characteristics

Table 2 presents the distribution of MDD-8 among different subpopulations across the survey year. Overall, the percentage of children receiving a diversified diet decreased from 53·1 % (95 % CI: 50·7 %, 55·5 %) in 2007 to 51·7 % (95 % CI: 49·6 %, 53·8 %) in 2012, then increased to 53·7 % (95 % CI: 51·8 %, 55·5 %) in 2017. While receiving a diversified diet fluctuated across most of the study factors, there was a gradual rise in MDD-8 among children aged 18–23 months, children of mothers in the youngest age group, and those in Java and Bali. On the contrary, the percentage of MDD-8 declined steadily among 6–11 months old children, children whose mothers working in agriculture, wealthier households and urban residents. Compared to MDD-8, the proportion of children meeting MDD-7 was higher across the three surveys but showed a similar pattern across the study variables (see online supplementary material, Supplemental Table 1).

**Table 2:** The proportion of minimum dietary diversity (MDD-8*) among children aged 6–23 months in Indonesia from 2007 to 2017

Characteristics	2007			2012			2017		
	*n*	%	95 % CI	*n*	%	95 % CI	*n*	%	95 % CI
Overall	2627	53·1	50·7, 55·5	2439	51·7	49·6, 53·8	2522	53·7	51·8, 55·5
Child factors									
Child’s age									
6–11 months	714	37·7	34·1, 41·5	581	34·5	31·5, 37·6	505	34·0	31·2, 36·9
12–17 months	1028	62·5	58·7, 66·2	898	58·8	55·3, 62·3	1047	62·1	58·8, 65·3
18–23 months	885	61·5	57·6, 65·2	960	64·6	61·2, 67·9	970	64·9	61·7, 67·9
Child’s sex									
Male	1399	52·6	49·6, 55·6	1250	50·7	47·8, 53·5	1309	52·3	49·9, 54·7
Female	1228	53·7	50·2, 57·1	1189	52·9	50·1, 55·6	1213	55·2	52·6, 57·8
Maternal factors									
Mother’s age									
< 25 years	684	47·1	42·7, 51·4	624	48·2	44·3, 52·1	564	54·5	50·6, 58·3
25–34 years	1430	56·6	53·7, 59·6	1292	53·0	50·3, 55·7	1335	54·0	51·5, 56·4
≥ 35 years	513	53·1	48·2, 57·9	523	53·5	49·0, 57·9	623	52·5	48·9, 56·0
Mother’s education									
None or incompleted primary school	282	40·0	34·5, 45·8	183	32·3	26·8, 38·3	127	35·0	28·8, 41·9
Completed primary school	1260	50·1	47·0, 53·2	1026	48·3	45·2, 51·4	946	48·1	45·4, 50·8
Completed secondary school	790	58·6	54·3, 62·8	793	58·8	55·1, 62·4	852	57·7	54·6, 60·7
Completed higher education	295	77·7	70·7, 83·5	437	64·5	58·9, 69·6	597	68·2	64·5, 71·7
Mother’s occupation									
Agricultural	357	43·0	37·5, 48·6	221	42·1	36·1, 48·4	152	37·8	32·1, 44·0
Non-agricultural	834	62·1	58·0, 66·1	977	58·0	54·5, 61·4	1082	59·3	56·4, 62·0
Not working	1436	50·9	47·7, 54·0	1241	49·3	46·4, 52·1	1288	52·0	49·4, 53·0
Mother’s access to media at least once a week									
None	369	36·9	32·2, 41·9	277	39·9	34·9, 45·1	313	47·1	42·4, 51·8
Any media	2258	56·8	54·3, 59·2	2162	53·7	51·5, 55·9	2522	54·8	52·8, 56·7
Mother’s involvement in decision-making									
Not involved	124	48·1	38·7, 57·6	176	42·8	35·9, 50·0	111	51·6	43·3, 59·8
Involved in any aspect	2503	53·3	50·9, 55·8	2263	52·5	50·4, 54·7	2411	53·8	51·9, 55·7
Paternal factors									
Father’s education									
None or incompleted primary school	275	42·5	37·0, 48·3	196	41·3	35·2, 47·8	160	41·2	35·3, 47·3
Completed primary school	1122	48·9	45·5, 52·2	958	47·6	44·4, 50·9	918	49·3	46·5, 52·1
Completed secondary school	894	58·9	54·8, 62·9	872	55·8	52·4, 59·2	945	55·5	52·4, 58·6
Completed higher education	336	72·1	64·6, 78·6	413	65·8	60·1, 71·0	499	68·1	63·9, 72·0
Father’s occupation									
Not working or agricultural	792	43·4	40·0, 46·9	524	40·7	37·1, 44·3	497	43·4	39·8, 47·1
Non-agricultural	1835	57·7	54·9, 60·5	1915	55·1	52·7, 57·6	2025	56·6	54·5, 58·6
Health care, household and community factors									
Number of antenatal care visits									
< 4	432	41·6	36·4, 47·0	265	36·0	31·5, 40·7	203	42·7	37·4, 48·2
≥ 4	2195	55·6	53·1, 58·1	2174	53·8	51·6, 56·1	2319	54·7	52·7, 56·6
Postnatal care									
No	307	44·0	38·6, 49·6	708	45·6	42·2, 49·1	768	52·5	49·3, 55·8
Yes	2320	54·1	51·6, 56·6	1724	54·6	51·9, 57·2	1737	54·1	51·8, 56·3
Number of children under 5 years of age within the household									
≤ 2	2501	53·4	50·9, 55·8	2332	52·4	50·2, 54·5	2409	53·9	52·0, 55·7
> 2	126	48·5	39·1, 58·0	107	36·8	29·2, 45·2	113	49·6	42·2, 57·0
Household wealth									
Poorest	368	40·4	35·5, 45·6	324	36·5	32·0, 41·2	392	41·0	36·9, 45·3
Poorer	464	46·2	41·1, 51·3	435	47·1	42·4, 51·9	460	49·2	45·0, 53·5
Middle	559	54·8	50·2, 59·3	540	51·7	47·2, 56·1	754	56·4	53·2, 59·6
Richer	612	57·9	53·6, 67·0	663	60·5	56·3, 64·5	408	63·4	58·8, 67·8
Richest	624	62·7	58·2, 67·1	477	57·9	53·4, 62·4	508	55·5	51·7, 59·3
Living residency									
Urban	1210	60·2	56·3, 64·1	1291	58·6	55·5, 61·6	1428	58·4	55·8, 60·9
Rural	1417	48·1	45·1, 51·1	1148	45·3	42·6, 48·1	1094	49·1	46·4, 51·8
Region									
Java and Bali	756	55·1	51·2, 58·9	753	55·2	51·8, 58·6	895	55·8	53·0, 58·5
Sumatera	871	54·6	50·9, 58·2	766	51·9	48·9, 54·9	721	55·9	52·4, 59·2
Kalimantan	272	51·1	46·1, 56·1	295	50·6	46·1, 55·1	236	54·9	49·1, 60·6
Sulawesi	404	47·8	43·6, 51·9	344	42·2	38·5, 46·1	327	44·9	40·2, 49·7
Eastern Indonesia	324	41·9	36·7, 47·3	281	35·2	31·1, 39·6	343	39·0	34·5, 43·7

*n*: weighted counts, % (95 % CI): weighted proportion and CI.

*Based on the WHO indicator of MDD of at least five of eight food groups in 2017^(9)^.

Figure 1 presents the trend of each food group’s consumption between 2007 and 2017. There was an increase in grains, roots and tubers, vitamin A-rich fruits and vegetables, and dairy products. Also, flesh foods and eggs consumption increased from 2012 to 2017. When we combined flesh foods and eggs, we also found a similar trend across the surveys. On the other hand, there was a steady decrease in children consuming legumes and nuts and breastmilk. The consumption of other types of fruits and vegetables dropped by almost half between 2007 and 2017.

**Fig. 2:**
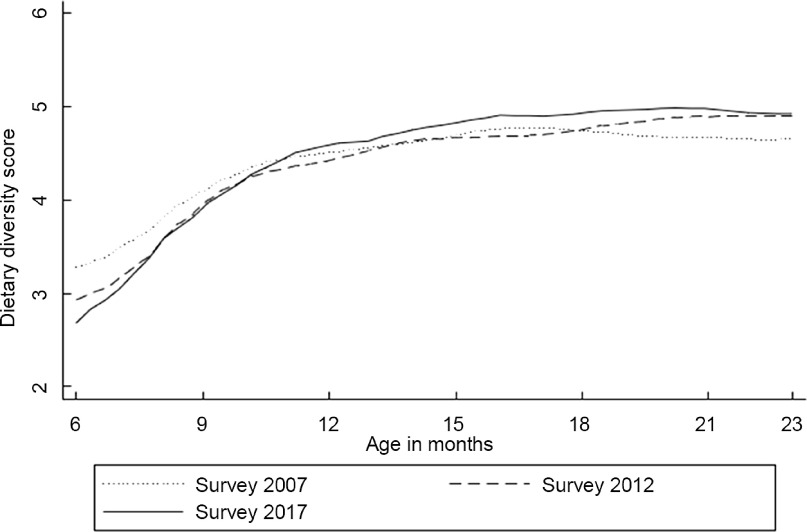
Mean of dietary diversity score by the child’s age in months

Figure 2 shows dietary diversity score patterns against the child’s age from local polynomial smoothing estimates. In general, dietary diversity increased gradually with increasing age. The 6-month dietary diversity differences varied between the surveys, with the 2017 IDHS survey having the lowest mean dietary diversity score. However, the differences became narrower by 10 months and remained small after that age. On average, children in all three surveys consumed four food groups when they reached 9 months, while five were achieved by the age of 18–23 months, except for the earliest survey.

### Factors associated with minimum dietary diversity

Table 3 presents unadjusted and adjusted OR of MDD-8 determinants by pooling data from 2007 to 2017. There was a dose–response association between the child’s age and MDD-8, with the highest odds among children aged 18–23 months (3·5 times). Level of education and access to media were among the maternal factors that were significantly related to MDD-8. Compared to mothers with no formal education, children whose mothers completed primary, secondary and higher education were 1·3–2·3 times more likely to consume a diversified diet. Mothers with weekly access to media had a 21 % greater odds of having children meeting the MDD-8 standard than mothers without weekly access to media. Fathers working in the non-agricultural sector were 1·3 times more likely to have children meeting MDD-8 than fathers with agricultural work or without a job. The odds of meeting the MDD-8 criterion was 23 % higher among children whose mothers had an ANC history of at least four visits than those with less than four visits. Compared to the poorest, children from the poorer, middle, richer and richest households had 18 %, 23 %, 48 % and 57 % greater odds of eating a diversified diet, respectively. Among the community-level factors, rural children had a 3 % lower odds of reaching MDD-8 than urban children. Using Java and Bali as the reference, children living in Sulawesi and Eastern Indonesia were 1·3 and 1·5 less likely to meet MDD-8, respectively. The associated factors were similar to those related to MDD-7, except maternal occupation and region (see online supplementary material, Supplemental Table 2).

**Table 3:** Factors associated with minimum dietary diversity (MDD-8*) among children aged 6–23 months in Indonesia showing unadjusted and adjusted† OR from 2007 to 2017

Variables	2007–2017					
	OR	95 % CI	*P*	AOR	95 % CI	*P*
Survey year						
2007	Ref			Ref		
2012	0·95	0·83, 1·08	0·404	0·78	0·68, 0·89	< 0·001
2017	1·02	0·91, 1·16	0·700	0·72	0·63, 0·83	< 0·001
Child factors						
Child’s age						
6–11 months	Ref			Ref		
12–17 months	2·87	2·57, 3·21	< 0·001	3·15	2·80, 3·54	< 0·001
18–23 months	3·20	2·86, 3·59	< 0·001	3·45	3·06, 3·89	< 0·001
Child’s sex						
Male	Ref			Ref		
Female	1·09	0·99, 1·19	0·073	1·05	0·95, 1·15	0·370
Maternal factors						
Mother’s age						
< 25 years	Ref			Ref		
25–34 years	1·22	1·09, 1·36	0·001	1·00	0·88, 1·12	0·909
≥ 35 years	1·15	1·00, 1·31	0·044	1·01	0·87, 1·17	0·902
Mother’s education						
None or incompleted primary school	Ref			Ref		
Completed primary school	1·68	1·43, 1·98	< 0·001	1·34	1·12, 1·61	0·001
Completed secondary school	2·46	2·06, 2·93	< 0·001	1·63	1·32, 2·00	< 0·001
Completed higher education	3·87	3·16, 4·75	< 0·001	2·25	1·74, 2·90	< 0·001
Mother’s occupation						
Agricultural	Ref			Ref		
Non-agricultural	2·08	1·78, 2·44	< 0·001	1·15	0·95, 1·40	0·141
Not working	1·45	1·25, 1·69	< 0·001	1·02	0·86, 1·22	0·794
Mother’s access to media at least once a week						
None	Ref			Ref		
Any media	1·76	1·55, 2·00	< 0·001	1·21	1·04, 1·39	0·012
Mother’s involvement in decision-making						
Not involved	Ref			Ref		
Involved in any aspect	1·31	1·08, 1·59	0·005	1·20	0·98, 1·47	0·084
Paternal factors						
Father’s education						
None or incompleted primary school	Ref			Ref		
Completed primary school	1·31	1·12, 1·55	0·001	0·94	0·78, 1·14	0·521
Completed secondary school	1·82	1·54, 2·14	< 0·001	0·99	0·80, 1·22	0·908
Completed higher education	2·99	2·43, 3·67	< 0·001	1·20	0·92, 1·55	0·173
Father’s occupation						
Not working or agricultural	Ref			Ref		
Non-agricultural	1·74	1·58, 1·93	< 0·001	1·25	1·10, 1·42	0·001
Health care, household and community factors						
Number of antenatal care visits						
< 4	Ref			Ref		
≥ 4	1·81	1·57, 2·07	< 0·001	1·23	1·06, 1·43	0·008
Postnatal care						
No	Ref					
Yes	1·27	1·14, 1·41	< 0·001			
Number of children under 5 years of age within the household						
≤ 2	Ref					
> 2	0·72	0·59, 0·89	0·002			
Household wealth						
Poorest	Ref			Ref		
Poorer	1·44	1·24, 1·68	< 0·001	1·18	1·00, 1·40	0·049
Middle	1·67	1·44, 1·93	< 0·001	1·23	1·04, 1·46	0·017
Richer	2·25	1·95, 2·60	< 0·001	1·48	1·25, 1·76	< 0·001
Richest	2·00	1·72, 2·32	< 0·001	1·57	1·31, 1·89	< 0·001
Living residency						
Urban	Ref			Ref		
Rural	0·95	0·94, 0·96	< 0·001	0·97	0·96, 0·99	< 0·001
Region						
Java and Bali	Ref			Ref		
Sumatera	0·95	0·85, 1·06	0·365	1·08	0·96, 1·22	0·208
Kalimantan	0·88	0·76, 1·01	0·064	1·07	0·92, 1·24	0·407
Sulawesi	0·66	0·58, 0·75	< 0·001	0·79	0·69, 0·90	0·001
Eastern Indonesia	0·51	0·44, 0·58	< 0·001	0·68	0·58, 0·80	< 0·001

OR, unadjusted OR; AOR, adjusted OR; Ref, reference; *P*, *P*-value.

* Based on the WHO indicator of MDD of at least five of eight food groups in 2017^(9)^.

† Independent variables adjusted for survey year, child factors (age and sex), maternal factors (age, education, occupation, access to media and involvement in decision-making), paternal factors (education and occupation), and health care, household and community factors (ANC visits, postnatal care, household wealth, number of children, living residency and region).

In addition, we ran logistic regression analyses for each survey period to help understand whether the determinants of MDD-8 were stable or shifting across time (see online supplementary material, Supplemental Table 3). Only children whose mothers completed higher education had relatively higher odds (2·6 times) of meeting MDD-8 in 2007. However, this trend changed in the later surveys, whereby children whose mothers completed at least primary school in 2012 and at least secondary school in 2017 were significantly more likely to meet MDD-8. Children of mothers with weekly access to media had a 21 % greater odds of meeting MDD-8 in 2007 than those whose mothers did not have weekly access to media. However, this trend shifted in 2012 and 2017, where the mother’s weekly access to media was not significantly associated with MDD-8. While the previous surveys did not find a significant association between parent’s occupation and MDD-8, children whose mothers and fathers worked in non-agricultural sectors were 45 % and 30 % more likely to meet MDD-8, respectively, in 2017. Among the health care-related factors, only ANC visits in 2017 and postnatal care in 2012 remained positively associated with MDD-8 in the adjusted analysis. Only children in the richest wealth quintile had greater odds (1·6 times) of meeting MDD-8 in 2007 than those in the poorest quintile. However, this pattern of household wealth changed in later surveys. Compared to children in the poorest quintile, children from the poorer, middle, richer and richest quintiles had greater odds of meeting MDD-8 in 2012, while children from the middle, richer and richest quintiles had greater odds of meeting MDD-8 in 2017. The odds of meeting the MDD-8 standard were 3 % lower in 2007 and 4 % lower in 2012 among children in rural areas than in urban areas. We did not find a significant relationship between residency and MDD-8 in 2017 in the adjusted analysis. In all three surveys, children living in Sulawesi and Eastern Indonesia remained less likely to meet MDD-8 than those living in Java and Bali.

### Interaction analyses on the factors associated with MDD-8

**Fig. 3:**
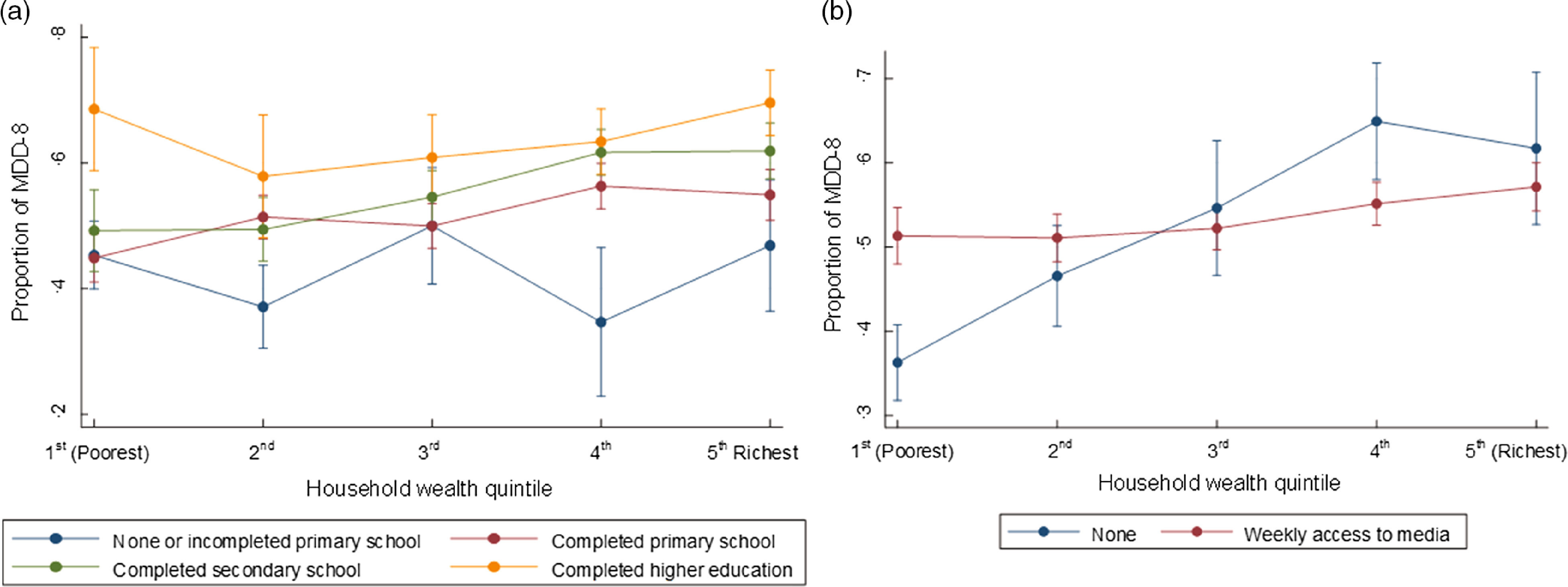
The combined effect of household wealth and mother’s education (*P* = 0·006) (a) and household wealth and mother’s access to media (*P* < 0·001) (b) on MDD-8 adjusted for other covariates. MDD, minimum dietary diversity

We found significant interactions between the mother’s education and household wealth (*P* = 0·006) and between the mother’s access to media and household wealth (*P* < 0·001) during our investigation on the model assessing factors related to MDD-8. From this model (see online supplementary material, Supplemental Table 4), we examined the combined effect of these factors on the percentage of children meeting MDD-8. As shown in Fig. 3, we found that the MDD-8 increases with household wealth among mothers with higher education but fluctuates among mothers with lower education. Additionally, the probability of children meeting MDD-8 increases with household wealth among mothers without weekly access to media but remains almost the same among mothers with weekly access to media.

## Discussion

### Main findings

This study analysed the MDD trends among infants and young children in Indonesia and the possible determinants at the child, parent, household, health care and community levels. Following a decline in MDD-8 among Indonesian infants and young children between 2007 and 2012, consuming at least five food groups increased from 2012 to 2017. The consumption of grains, roots and tubers, flesh foods, eggs, and dairy products rose over the past decade. Conversely, legumes and nuts, breastmilk, and other fruits and vegetables depicted a declining trend. Using the pooled data from 2007 to 2017, we found that the odds of meeting MDD-8 decreased with the survey year. The adjusted analysis showed an association between MDD-8 and the child’s age, maternal educational level, maternal access to media, paternal occupation, ANC visit, household wealth index, residency, and region. However, the child’s age, maternal education and household wealth were among the factors consistently associated with MDD-8 across the three surveys.

### Trends of minimum dietary diversity and food group consumption

A study conducted in Nepal found a similar dietary diversity trend, with 32 % of children in 2006 meeting the MDD-7 criterion, decreasing to 28 % in 2011 before rising to 36 % in 2014. The pattern was not influenced by improvements in maternal education and access to health care over time, suggesting broader issues were important in Nepal^(19)^. Economic changes may affect the dietary habits and dietary quality in a population. The deterioration in dietary diversity between 2007 and 2012 might be affected by the prolonged effect of the 2007/2008 global food crisis that significantly hit the Indonesian households, resulting in the consumption that focused on sustaining dietary energy intake but a less varied diet^(29)^. As part of the responses to the crisis, the Indonesian government made several efforts to improve community nutrition through the Rice for the Poor programme^(29)^, national policy on dietary diversification (e.g. the 2012 Law on Food of the Republic of Indonesia^(30)^ and the 2014 Balanced Nutrition Guideline^(31)^) and food price subsidies^(32)^. There was also a tremendous increase in small and medium enterprises and the female labour force participation to improve the household purchasing power^(32)^. These changes might have reversed adequate child dietary diversity in 2017 by about 53 %, to a level similar to the pre-crisis situation. However, after accounting for other study variables in the adjusted analysis, we found that children were less likely to meet MDD-8 in 2012 and 2017 compared to 2007. The results suggest that different characteristics of the children might influence MDD-8.

Similar to our findings, there was an upward pattern in the consumption of eggs, dairy products, and vitamin A-rich fruits and vegetables in Cambodia^(33)^. Conversely, there was a decrease in grains, roots and tubers in Cambodia 2005–2014^(33)^ and Nepal 2001–2014^(19)^. We also identified a trend difference for legumes, nuts, and other fruits and vegetables between Indonesia and Cambodia, where these food groups increased slightly^(33)^.

Our findings indicate that infants and young children in Indonesia consumed higher energy and fat intakes from animal source foods but decreased plant-based foods consumption. There has been a major shift in how food is produced and consumed in Indonesia. The Rice for the Poor programme distributed to all regions during the crisis replaced the local staple food (e.g. maize in East Nusa Tenggara and sago in Maluku) with rice^(32)^. Even young people considered rice to be more prestigious than the local food. Since rice has dominated the population’s consumption and served as the primary source of vegetable protein^(32)^, this might underline the downward trend in legumes, nuts and other vegetable consumption. There was also a lifestyle change marked by women’s participation in the workforce resulting in the dietary shift to eating outside the house and consuming fast food^(32)^. The increase in women’s participation in the workforce also led to mothers being unable to breastfeed their children optimally, as shown in the declining trend of current breast-feeding status.

### Factors associated with minimum dietary diversity

Our study findings are similar to previous national-level studies in other countries, suggesting a significant association between the child’s age and dietary diversity^(18,27,28)^. A potential explanation for older infants to consume a more diversified diet could be their readiness to accept food with various forms (e.g. textures and tastes) and their greater familiarity with food than younger infants^(34)^. In Indonesia, food culture may also affect child feeding practices. As soon as infants started eating, rice was the primary preference because it was considered precious. Some infants below 1 year of age only received rice porridge and water from boiled vegetables, while family food would be given after 1 year^(35)^. In some regions, young infants were not allowed to consume fish^(35,36)^ and peas^(36)^.

Consistent with earlier studies in Indonesia^(15,17)^ and in other low- and middle-income countries^(18,24,27,28)^, we found that mothers with higher educational levels tend to feed their children with a more diversified diet. Highly educated mothers might have more information and better understand educational messages, and they might also have learnt about child feeding at school^(37)^. The trend showed a much smaller influence of mother’s education on MDD-8 in 2007, where only children whose mothers completed a higher degree were more likely to meet MDD-8. The effect increased in the subsequent surveys. Children of mothers with at least primary education in 2012 and at least secondary education in 2017 were more likely to meet MDD-8 than children of mothers without formal education. The improvement noticed can be explained by the improved quality of the Indonesian education and health systems^(38)^.

We also found a significant relationship between mother’s access to any media (i.e. newspaper, magazine, radio or television at least once a week) and MDD-8, as also observed in Ethiopia^(26)^ and India^(28)^, but not in Myanmar^(17)^. Mass media was considered a reliable source of information that could be influencing behaviour^(39)^. For example, the Indonesian Ministry of Health has circulated the Ten Balanced Nutrition Messages via different mass media, including ‘Be thankful and eat a variety of food’, a key message for improving dietary diversity^(40)^. The stratified analysis showed that mother’s access to media was associated with MDD-8 only in 2007 but not in later surveys. This finding indicates that the delivery of nutrition information has shifted from printed media, radio and television to the internet. Moreover, the use of printed media and radio has declined sharply over time^(41)^.

In contrast to our study, the father’s occupation was not a determinant of child dietary diversity in Myanmar and Cambodia^(17)^. Our finding suggests that working in agricultural fields might not generate sufficient income. While smallholder farmers dominate Indonesian agriculture, a study in East Kalimantan showed that paddy and non-paddy farming jobs only contributed to 42·3 % and 50·7 % of household income, respectively^(42)^. In a stratified analysis, paternal occupation and MDD-8 were only linked in 2017, but not in the earlier surveys, suggesting a disparity in MDD-8 among children based on the paternal work in the latest survey.

Previous Indonesian studies reported a dose–response relationship between household wealth and dietary diversity^(16,17)^. In our study, children from the wealthiest families had the greatest chance of experiencing a diverse diet, followed by children from progressively poorer families. More affluent families could afford a range of animal and plant source foods^(16)^. While the 2007 survey only showed a significant relationship between the richest household and MDD-8, the trend shows a more substantial effect of household wealth in 2012 and 2017. MDD-8 was associated with middle, richer and richest households indicating that a varied diet was more accessible to a broader population.

ANC visit frequency was significantly associated with child dietary diversity in our study and studies from Nepal^(19)^ and India^(28)^. However, we only found a significant relationship in 2017 but not in earlier surveys in our stratified analysis. Maternal visits to ANC reflected maternal access to services related to health and nutrition. For example, mothers might obtain information about appropriate child feeding through a counselling session during ANC^(16)^. Our results indicate that ANC should continue providing counselling and education related to infant and young child feeding practices. Besides, barriers to ANC service quality, such as the long queue and short counselling time^(43)^, and insufficient knowledge and training of the health workers^(44)^, should be eliminated.

In agreement with our findings, an earlier study in China^(45)^ reported a more diversified diet among urban residents than rural residents. Similarly, in Cambodia and Myanmar, the likelihood of infant and young children reaching MDD varied across geographical regions^(17)^. Less access to food and lower economic status might explain the higher risk of low dietary diversity among children from rural areas^(45)^. Variations of food traditions or the availability of foods^(46)^ might also have contributed to the geographical differences. However, unlike the previous surveys, the trend analysis showed no significant link between residence and MDD-8 in 2017, implying that living in urban areas does not necessarily mean eating a diverse diet.

### Interactions between household wealth and mother’s education and between household wealth and mother’s access to media on minimum dietary diversity

We found interactions between household wealth and mother’s education and between household wealth and mother’s access to media on MDD-8. These interactions showed a high proportion of children meeting MDD-8 across household wealth categories among highly educated mothers but not among mothers with lower levels of education. This finding suggests socio-economic inequalities in MDD-8 in Indonesia, as reported in a previous study in low- and middle-income countries^(47)^. Additionally, we found a high proportion of children meeting MDD-8 across household wealth groups among mothers without weekly access to media. However, the proportion of children meeting MDD-8 was steady across household wealth groups among mothers with weekly access to media. This finding indicates that maternal access to media may reduce the gap in consumption of a diversified diet between rich- and low-income families.

### Strengths and limitations

This paper is the first to report dietary diversity trends and determinants based on the nationally representative samples from 2007 to 2017 in Indonesia using the most updated MDD indicator (MDD-8)^(9)^. Moreover, we also presented the results for the previously used indicator (MDD-7)^(10)^. The statistical analysis was adjusted for the DHS complex sampling design by applying sampling weights, clustering and stratification using ‘svy’ survey commands in Stata. Limitations are that the 2007 IDHS collected flesh foods and eggs data as one item, so we could not assess their consumption separately. Our study might also have recall bias, because food intake data were collected based on maternal recall. Another limitation is the inability to infer causality because of the cross-sectional survey design.

### Policy implications

This study identifies determinants of dietary diversity and offers opportunities for intervention. Notably, from 2007 to 2017, children of less-educated mothers and poor households were less likely to eat a diversified diet. Thus, prioritising maternal and household factors associated with MDD-8 might be the most promising way to reduce the burden of inadequate dietary diversity and socio-economic inequalities. Furthermore, enhancing access to nutrition-related information from various resources, such as the internet and health facilities, may help improve this outcome since the children of some poor mothers can meet MDD-8 when they have access to appropriate information. Nutrition education^(48)^ and behaviour change communication activities^(49)^ can improve infant and young child feeding practices. Such interventions should target mothers from pregnancy^(49)^, particularly those of lower socio-economic status, those living in rural and underdeveloped regions where access to information is limited. Furthermore, the country will require sufficient resources to train health practitioners and community health workers^(48,49)^ to deliver high-quality counselling and education for all women throughout the 1000 d of life. We should integrate nutrition with agriculture activities to improve household dietary diversity and the nutritional status of infants and young children^(48,50)^.

## Conclusion

Our findings showed that the overall prevalence of MDD has stagnated from 2007 to 2017. During this period, consuming a diversified diet was associated with a child’s age, mother’s educational attainment, mother’s access to media, father’s occupation, ANC visits, household wealth quintiles, living residency and region.

## Supplementary material

For supplementary material accompanying this paper visit https://doi.org/10.1017/S1368980021004559. click here to view supplementary material
