# Supplementary material for: Trends and determinants of minimum dietary diversity among children aged 6–23 months: a pooled analysis of Indonesia Demographic and Health Surveys from 2007 to 2017
Source: Public Health Nutr. 2021 Nov 8;25(7):1956–67. doi: 10.1017/S1368980021004559 (PMC9991623; doi:10.1017/S1368980021004559)
Supplement: Supplementary file 1 [file S1368980021004559sup.zip › S1368980021004559sup002.docx]

# Supplementary material

**Supplemental Table 1.** The proportion of minimum dietary diversity (MDD-7^a^) among children aged 6-23 months in Indonesia from 2007 to 2017

| Characteristics | 2007 | | 2012 | | 2017 | |
| --- | --- | --- | --- | --- | --- | --- |
|  | n | % (95% CI) | n | % (95% CI) | n | % (95% CI) |
| Overall | 2925 | 58.5 (56.1-60.9) | 2766 | 57.2 (55.0-59.4) | 2849 | 59.5 (57.6-61.4) |
| **Child factors** |  |  |  |  |  |  |
| Child’s age |  |  |  |  |  |  |
| 6-11 months | 787 | 40.2 (36.5-44.0) | 645 | 37.2 (34.1-40.3) | 577 | 38.3 (35.4-41.3) |
| 12-17 months | 1097 | 66.4 (62.6-70.1) | 1004 | 63.5 (60.0-67.0) | 1160 | 67.2 (63.8-70.3) |
| 18-23 months | 1041 | 71.9 (68.2-75.4) | 1117 | 74.1 (70.9-77.1) | 1112 | 73.2 (70.2-76.0) |
| Child’s sex |  |  |  |  |  |  |
| Male | 1561 | 58.7 (55.6-61.7) | 1428 | 56.0 (53.1-58.8) | 1494 | 58.3 (55.8-60.6) |
| Female | 1364 | 58.3 (54.8-61.7) | 1338 | 58.5 (55.6-61.2) | 1355 | 60.9 (58.3-63.5) |
| **Maternal factors** |  |  |  |  |  |  |
| Mother’s age |  |  |  |  |  |  |
| <24 years | 777 | 53.2 (48.6-57.6) | 715 | 53.9 (50.0-57.9) | 635 | 60.8 (57.0-64.5) |
| 25-34 years | 1588 | 62.3 (59.4-65.1) | 1473 | 59.0 (56.2-61.6) | 1498 | 59.1 (56.7-61.5) |
| >35 years | 560 | 56.9 (52.1-61.6) | 578 | 57.1 (52.6-61.5) | 716 | 59.3 (57.7-61.4) |
| Mother’s education |  |  |  |  |  |  |
| None or incompleted primary school | 315 | 44.3 (38.9-49.9) | 212 | 36.0 (30.4-42.0) | 143 | 42.3 (34.9-50.1) |
| Completed primary school | 1401 | 54.8 (51.6-57.9) | 1161 | 52.6 (49.4-55.8) | 1089 | 54.1 (51.3-56.8) |
| Completed secondary school | 888 | 66.7 (62.3-70.8) | 898 | 66.5 (62.8-69.9) | 950 | 63.4 (60.2-66.4) |
| Completed higher education | 321 | 82.1 (75.1-87.4) | 495 | 70.8 (65.2-75.8) | 667 | 73.5 (70.0-76.7) |
| Mother’s occupation |  |  |  |  |  |  |
| Agricultural | 392 | 48.1 (42.8-53.6) | 255 | 46.7 (40.4-53.0) | 179 | 43.8 (37.8-50.0) |
| Non-agricultural | 932 | 67.5 (63.3-71.4) | 1117 | 65.5 (62.1-68.9) | 1240 | 66.7 (63.9-69.4) |
| Not working | 1601 | 56.4 (53.2-59.5) | 1394 | 53.5 (50.6-56.3) | 1430 | 56.7 (54.1-59.3) |
| Mother’s access to media at least once a week |  |  |  |  |  |  |
| None | 408 | 40.5 (35.6-45.5) | 323 | 43.6 (38.4-48.9) | 358 | 50.8 (46.1-55.5) |
| Any media | 2517 | 62.6 (60.1-65.0) | 2443 | 59.4 (57.1-61.7) | 2491 | 61.0 (59.0-62.9) |
| Mother’s involvement in decision making |  |  |  |  |  |  |
| Not involved | 142 | 52.8 (43.3-62.2) | 209 | 48.5 (41.5-55.6) | 125 | 60.0 (51.7-67.7) |
| Involved in any aspect | 2783 | 58.8 (56.3-61.2) | 2557 | 58.0 (55.8-60.1) | 2724 | 59.5 (57.6-61.4) |
| **Paternal factors** |  |  |  |  |  |  |
| Father’s education |  |  |  |  |  |  |
| None or incompleted primary school | 310 | 46.3 (40.7-52.0) | 231 | 47.8 (41.6-54.1) | 182 | 47.1 (40.6-53.8) |
| Completed primary school | 1254 | 54.4 (51.0-57.7) | 1075 | 51.9 (48.6-55.2) | 1033 | 54.4 (51.6-57.2) |
| Completed secondary school | 993 | 64.8 (60.6-68.7) | 993 | 62.5 (59.2-65.7) | 1081 | 62.4 (59.2-65.5) |
| Completed higher education | 368 | 77.9 (70.8-83.6) | 467 | 72.0 (66.5-76.8) | 553 | 73.6 (69.7-77.2) |
| Father’s occupation |  |  |  |  |  |  |
| Not working or agricultural | 881 | 48.0 (44.3-51.6) | 597 | 44.9 (41.3-48.5) | 562 | 47.6 (44.0-51.3) |
| Non-agricultural | 2044 | 63.6 (60.7-66.3) | 2169 | 60.9 (58.4-63.4) | 2287 | 62.9 (60.8-64.9) |
| **Health care, household, and community factors** |  |  |  |  |  |  |
| Number of antenatal care visits |  |  |  |  |  |  |
| <4 | 484 | 46.6 (41.3-51.9) | 316 | 41.4 (36.7-46.2) | 229 | 48.0 (42.4-53.6) |
| >4 | 2441 | 61.1 (58.5-63.6) | 2450 | 59.3 (57.0-61.5) | 2620 | 60.6 (58.6-62.5) |
| Postnatal care |  |  |  |  |  |  |
| No | 343 | 49.9 (44.2-55.6) | 828 | 51.1 (47.6-54.5) | 768 | 52.5 (49.3-55.8) |
| Yes | 2582 | 59.5 (57.0-62.0) | 1930 | 60.0 (57.3-62.7) | 1737 | 54.1 (51.8-56.3) |
| Number of children under five within the household |  |  |  |  |  |  |
| <2 | 2780 | 58.7 (56.2-61.2) | 2639 | 57.8 (55.6-60.0) | 2718 | 59.7 (57.8-61.6) |
| >2 | 145 | 55.0 (46.0-63.7) | 127 | 42.7 (34.7-51.0) | 131 | 55.0 (47.7-62.1) |
| Household wealth |  |  |  |  |  |  |
| Poorest | 764 | 46.6 (42.6-50.6) | 427 | 42.8 (38.4-47.4) | 307 | 46.5 (41.4-51.8) |
| Poorer | 820 | 60.4 (56.4-64.3) | 469 | 51.6 (46.6-56.5) | 314 | 48.8 (43.8-53.8) |
| Middle | 619 | 62.1 (57.1-66.9) | 659 | 59.8 (55.2-64.3) | 561 | 58.0 (54.1-61.8) |
| Richer | 365 | 66.7 (60.1-72.6) | 676 | 65.5 (61.0-69.6) | 966 | 66.8 (63.7-69.8) |
| Richest | 357 | 71.3 (64.2-77.5) | 535 | 62.4 (57.7-66.8) | 701 | 61.3 (57.8-64.7) |
| Living residency |  |  |  |  |  |  |
| Urban | 1336 | 65.6 (61.6-69.3) | 1450 | 64.0 (60.8-67.1) | 1608 | 64.9 (62.3-67.5) |
| Rural | 1589 | 53.6 (50.4-56.7) | 1316 | 50.7 (47.8-53.6) | 1241 | 54.3 (51.5-57.0) |
| Region |  |  |  |  |  |  |
| Java and Bali | 819 | 59.8 (55.7-63.7) | 819 | 59.8 (56.3-63.3)\ | 984 | 61.2 (58.3-63.9) |
| Sumatera | 975 | 60.9 (57.3-64.4) | 874 | 58.7 (55.7-61.7) | 801 | 61.7 (58.3-63.9) |
| Kalimantan | 299 | 57.4 (52.4-62.2) | 328 | 55.8 (51.4-60.2) | 273 | 61.5 (55.4-67.3) |
| Sulawesi | 459 | 54.1 (49.9-58.3) | 407 | 49.5 (45.6-53.3) | 386 | 53.4 (48.7-58.0) |
| Eastern Indonesia | 373 | 48.0 (42.5-53.5) | 338 | 41.3 (36.7-46.1) | 405 | 45.4 (40.4-50.4) |

^a^Based upon the WHO indicator of minimum dietary diversity of at least 4 of 7 food groups in 2008 ^(14)^

n: weighted counts, % (95% CI): weighted proportion and confidence intervals

**Supplemental Table 2.** Factors associated with minimum dietary diversity (MDD-7^a^) among children aged 6-23 months in Indonesia showing unadjusted and adjustedb odds ratios from 2007 to 2017

| Variables | 2007-2017 | | | | | |
| --- | --- | --- | --- | --- | --- | --- |
|  | OR | 95% CI | p | AOR | 95% CI | p |
| Survey year |  |  |  |  |  |  |
| 2007 | Ref |  |  | Ref |  |  |
| 2012 | 0.95 | 0.83-1.08 | 0.419 | 0.76 | 0.66-0.87 | <0.001 |
| 2017 | 1.04 | 0.93-1.18 | 0.509 | 0.71 | 0.62-0.83 | <0.001 |
| **Child factors** |  |  |  |  |  |  |
| Child’s age |  |  |  |  |  |  |
| 6-11 months | Ref |  |  | Ref |  |  |
| 12-17 months | 3.06 | 2.73-3.42 | <0.001 | 3.43 | 3.04-3.88 | <0.001 |
| 18-23 months | 4.33 | 3.84-4.88 | <0.001 | 4.85 | 4.28-5.49 | <0.001 |
| Child’s sex |  |  |  |  |  |  |
| Male | Ref |  |  | Ref |  |  |
| Female | 1.07 | 0.98-1.17 | 0.146 | 1.03 | 0.93-1.13 | 0.580 |
| **Maternal factors** |  |  |  |  |  |  |
| Mother’s age |  |  |  |  |  |  |
| <24 years | Ref |  |  | Ref |  |  |
| 25-34 years | 1.20 | 1.07-1.34 | 0.001 | 0.95 | 0.84-1.07 | 0.400 |
| >35 years | 1.10 | 0.96-1.25 | 0.164 | 0.94 | 0.81-1.09 | 0.382 |
| Mother’s education |  |  |  |  |  |  |
| None or incompleted primary school | Ref |  |  | Ref |  |  |
| Completed primary school | 1.68 | 1.43-1.97 | <0.001 | 1.33 | 1.11-1.59 | 0.002 |
| Completed secondary school | 2.71 | 2.28-3.23 | <0.001 | 1.74 | 1.41-2.14 | <0.001 |
| Completed higher education | 4.15 | 3.37-5.11 | <0.001 | 2.24 | 1.72-2.91 | <0.001 |
| Mother’s occupation |  |  |  |  |  |  |
| Agricultural | Ref |  |  | Ref |  |  |
| Non-agricultural | 2.27 | 1.94-2.67 | <0.001 | 1.25 | 1.03-1.52 | 0.028 |
| Not working | 1.43 | 1.23-1.66 | <0.001 | 0.99 | 0.83-1.19 | 0.929 |
| Mother’s access to media at least once a week |  |  |  |  |  |  |
| None | Ref |  |  | Ref |  |  |
| Any media | 1.93 | 1.70-2.19 | <0.001 | 1.33 | 1.14-1.54 | <0.001 |
| Mother’s involvement in decision making |  |  |  |  |  |  |
| Not involved | Ref |  |  | Ref |  |  |
| Involved in any aspect | 1.29 | 1.06-1.56 | 0.011 | 1.17 | 0.94-1.44 | 0.153 |
| **Paternal factors** |  |  |  |  |  |  |
| Father’s education |  |  |  |  |  |  |
| None or incompleted primary school | Ref |  |  | Ref |  |  |
| Completed primary school | 1.30 | 1.10-1.53 | 0.002 | 0.90 | 0.75-1.09 | 0.278 |
| Completed secondary school | 1.93 | 1.64-2.27 | <0.001 | 0.99 | 0.80-1.21 | 0.905 |
| Completed higher education | 3.21 | 2.60-3.97 | <0.001 | 1.22 | 0.94-1.58 | 0.135 |
| Father’s occupation |  |  |  |  |  |  |
| Not working or agricultural | Ref |  |  | Ref |  |  |
| Non-agricultural | 1.88 | 1.70-2.08 | <0.001 | 1.36 | 1.19-1.54 | <0.001 |
| **Health care, household, and community factors** |  |  |  |  |  |  |
| Number of antenatal care visits |  |  |  |  |  |  |
| <4 | Ref |  |  | Ref |  |  |
| >4 | 1.84 | 1.61-2.10 | <0.001 | 1.22 | 1.05-1.42 | 0.010 |
| Postnatal care |  |  |  |  |  |  |
| No | Ref |  |  |  |  |  |
| Yes | 1.25 | 1.13-1.40 | <0.001 |  |  |  |
| Number of children under five within the household |  |  |  |  |  |  |
| <2 | Ref |  |  |  |  |  |
| >2 | 0.73 | 0.60-0.90 | 0.002 |  |  |  |
| Household wealth |  |  |  |  |  |  |
| Poorest | Ref |  |  | Ref |  |  |
| Poorer | 1.49 | 1.28-1.73 | <0.001 | 1.21 | 1.02-1.43 | 0.027 |
| Middle | 1.80 | 1.56-2.09 | <0.001 | 1.30 | 1.09-1.54 | 0.003 |
| Richer | 2.37 | 2.04-2.75 | <0.001 | 1.51 | 1.26-1.81 | <0.001 |
| Richest | 2.08 | 1.79-2.42 | <0.001 | 1.59 | 1.32-1.92 | <0.001 |
| Living residency |  |  |  |  |  |  |
| Urban | Ref |  |  | Ref |  |  |
| Rural | 0.95 | 0.94-0.96 | <0.001 | 0.97 | 0.96-0.99 | <0.001 |
| Region |  |  |  |  |  |  |
| Java and Bali | Ref |  |  | Ref |  |  |
| Sumatera | 1.01 | 0.90-1.13 | 0.902 | 1.18 | 1.04-1.33 | 0.011 |
| Kalimantan | 0.91 | 0.79-1.06 | 0.230 | 1.15 | 0.98-1.35 | 0.090 |
| Sulawesi | 0.72 | 0.64-0.82 | <0.001 | 0.89 | 0.77-1.03 | 0.125 |
| Eastern Indonesia | 0.54 | 0.46-0.62 | <0.001 | 0.75 | 0.64-0.88 | 0.001 |

OR: unadjusted odds ratios; AOR: adjusted odds ratios; Ref: reference; p: p-value

^a^Based upon the WHO indicator of minimum dietary diversity of at least 4 of 7 food groups in 2017 ^(14)^

^b^Independent variables adjusted for: survey year, child factors (age, sex), maternal factors (age, education, occupation, access to media, involvement in decision-making), paternal factors (education, occupation), and health care, household and community factors (ANC visits, postnatal care, household wealth, number of children, living residency, and region)

**Supplemental Table 3.** Factors associated with minimum dietary diversity (MDD-8^a^) among children aged 6-23 months in Indonesia showing adjusted^b^ odds ratios in 2007, 2012, and 2017

| Variables | 2007 | | | 2012 | | | 2017 | | |
| --- | --- | --- | --- | --- | --- | --- | --- | --- | --- |
|  | AOR | 95% CI | p | AOR | 95% CI | p | AOR | 95% CI | p |
| **Child factors** |  |  |  |  |  |  |  |  |  |
| Child’s age |  |  |  |  |  |  |  |  |  |
| 6-11 months | Ref |  |  | Ref |  |  | Ref |  |  |
| 12-17 months | 2.95 | 2.36-3.68 | <0.001 | 3.02 | 2.46-3.70 | <0.001 | 3.66 | 3.00-4.48 | <0.001 |
| 18-23 months | 2.77 | 2.20-3.49 | <0.001 | 3.86 | 3.14-4.75 | <0.001 | 4.10 | 3.38-4.95 | <0.001 |
| Child’s sex |  |  |  |  |  |  |  |  |  |
| Male | Ref |  |  | Ref |  |  | Ref |  |  |
| Female | 1.04 | 0.86-1.25 | 0.704 | 1.03 | 0.87-1.21 | 0.770 | 1.07 | 0.92-1.25 | 0.368 |
| **Maternal factors** |  |  |  |  |  |  |  |  |  |
| Mother’s age |  |  |  |  |  |  |  |  |  |
| <24 years | Ref |  |  | Ref |  |  | Ref |  |  |
| 25-34 years | 1.32 | 1.07-1.62 | 0.010 | 0.93 | 0.76-1.13 | 0.449 | 0.79 | 0.65-0.96 | 0.020 |
| >35 years | 1.28 | 0.96-1.69 | 0.092 | 1.05 | 0.81-1.36 | 0.707 | 0.76 | 0.61-0.96 | 0.020 |
| Mother’s education |  |  |  |  |  |  |  |  |  |
| None or incompleted primary school | Ref |  |  | Ref |  |  | Ref |  |  |
| Completed primary school | 1.27 | 0.95-1.69 | 0.101 | 1.46 | 1.04-2.04 | 0.028 | 1.35 | 0.97-1.89 | 0.076 |
| Completed secondary school | 1.34 | 0.95-1.89 | 0.099 | 1.79 | 1.24-2.59 | 0.002 | 1.87 | 1.31-2.68 | 0.001 |
| Completed higher education | 2.64 | 1.57-4.42 | <0.001 | 2.01 | 1.28-3.16 | 0.002 | 2.68 | 1.78-4.03 | <0.001 |
| Mother’s occupation |  |  |  |  |  |  |  |  |  |
| Agricultural | Ref |  |  | Ref |  |  | Ref |  |  |
| Non-agricultural | 1.31 | 0.96-1.79 | 0.083 | 1.04 | 0.74-1.45 | 0.823 | 1.45 | 1.03-2.04 | 0.031 |
| Not working | 1.11 | 0.83-1.49 | 0.483 | 0.90 | 0.65-1.24 | 0.506 | 1.36 | 0.98-1.87 | 0.064 |
| Mother’s access to media at least once a week |  |  |  |  |  |  |  |  |  |
| None | Ref |  |  | Ref |  |  | Ref |  |  |
| Any media | 1.61 | 1.26-2.07 | <0.001 | 1.09 | 0.84-1.42 | 0.518 | 1.02 | 0.82-1.27 | 0.877 |
| Mother’s involvement in decision making |  |  |  |  |  |  |  |  |  |
| Not involved | Ref |  |  | Ref |  |  | Ref |  |  |
| Involved in any aspect | 1.12 | 0.73-1.74 | 0.603 | 1.32 | 0.97-1.79 | 0.077 | 1.05 | 0.74-1.51 | 0.776 |
| **Paternal factors** |  |  |  |  |  |  |  |  |  |
| Father’s education |  |  |  |  |  |  |  |  |  |
| None or incompleted primary school | Ref |  |  | Ref |  |  | Ref |  |  |
| Completed primary school | 1.03 | 0.75-1.41 | 0.867 | 0.87 | 0.62-1.23 | 0.438 | 0.94 | 0.70-1.25 | 0.652 |
| Completed secondary school | 1.22 | 0.86-1.73 | 0.262 | 0.92 | 0.63-1.35 | 0.671 | 0.90 | 0.65-1.25 | 0.534 |
| Completed higher education | 1.20 | 0.72-2.02 | 0.484 | 1.06 | 0.67-1.69 | 0.803 | 1.29 | 0.87-1.91 | 0.215 |
| Father’s occupation |  |  |  |  |  |  |  |  |  |
| Not working or agricultural |  |  |  |  |  |  | Ref |  |  |
| Non-agricultural |  |  |  |  |  |  | 1.30 | 1.06-1.60 | 0.012 |
| **Health care, household, and community factors** |  |  |  |  |  |  |  |  |  |
| Number of antenatal care visits |  |  |  |  |  |  |  |  |  |
| <4 |  |  |  |  |  |  | Ref |  |  |
| >4 |  |  |  |  |  |  | 1.32 | 1.02-1.71 | 0.037 |
| Postnatal care |  |  |  |  |  |  |  |  |  |
| No |  |  |  | Ref |  |  |  |  |  |
| Yes |  |  |  | 1.29 | 1.05-1.60 | 0.013 |  |  |  |
| Number of children under five within the household |  |  |  |  |  |  |  |  |  |
| <2 |  |  |  |  |  |  |  |  |  |
| >2 |  |  |  |  |  |  |  |  |  |
| Household wealth |  |  |  |  |  |  |  |  |  |
| Poorest | Ref |  |  | Ref |  |  | Ref |  |  |
| Poorer | 1.19 | 0.88-1.62 | 0.260 | 1.41 | 1.05-1.88 | 0.022 | 1.22 | 0.94-1.58 | 0.134 |
| Middle | 1.31 | 0.99-1.72 | 0.057 | 1.49 | 1.10-2.00 | 0.009 | 1.36 | 1.06-1.73 | 0.014 |
| Richer | 1.30 | 0.95-1.78 | 0.102 | 1.87 | 1.39-2.51 | <0.001 | 1.66 | 1.24-2.23 | 0.001 |
| Richest | 1.59 | 1.16-2.19 | 0.004 | 2.20 | 1.60-3.03 | <0.001 | 1.36 | 1.04-1.77 | 0.023 |
| Living residency |  |  |  |  |  |  |  |  |  |
| Urban | Ref |  |  | Ref |  |  |  |  |  |
| Rural | 0.97 | 0.95-0.99 | 0.013 | 0.96 | 0.94-0.98 | 0.001 |  |  |  |
| Region |  |  |  |  |  |  |  |  |  |
| Java and Bali |  |  |  | Ref |  |  | Ref |  |  |
| Sumatera |  |  |  | 0.95 | 0.80-1.16 | 0.612 | 1.13 | 0.93-1.38 | 0.206 |
| Kalimantan |  |  |  | 0.97 | 0.76-1.25 | 0.838 | 1.19 | 0.90-1.56 | 0.218 |
| Sulawesi |  |  |  | 0.66 | 0.53-0.83 | <0.001 | 0.71 | 0.55-0.90 | 0.004 |
| Eastern Indonesia |  |  |  | 0.62 | 0.48-0.81 | <0.001 | 0.58 | 0.45-0.75 | <0.001 |

AOR: adjusted odds ratios; Ref: reference; p: p-value

^a^Based upon the WHO indicator of minimum dietary diversity of at least 5 of 8 food groups in 2017 ^(13)^

^b^Independent variables adjusted for: survey year, child factors (age, sex), maternal factors (age, education, occupation, access to media, involvement in decision-making), paternal factors (education, occupation), and health care, household and community factors (ANC visits, postnatal care, household wealth, number of children, living residency, and region)

**Supplemental Table 4.** Factors associated with minimum dietary diversity (MDD-8^a^) among children aged 6-23 months in Indonesia adjusted by significant interactions, showing unadjusted and adjusted^b^ odds ratios from 2007 to 2017

| Variables | 2007-2017 | | | | | |
| --- | --- | --- | --- | --- | --- | --- |
|  | OR | 95% CI | p | AOR | 95% CI | p |
| Survey year |  |  |  |  |  |  |
| 2007 | Ref |  |  | Ref |  |  |
| 2012 | 0.95 | 0.83-1.08 | 0.404 | 0.77 | 0.67-0.88 | <0.001 |
| 2017 | 1.02 | 0.91-1.16 | 0.700 | 0.70 | 0.61-0.81 | <0.001 |
| Child factors |  |  |  |  |  |  |
| Child’s age |  |  |  |  |  |  |
| 6-11 months | Ref |  |  | Ref |  |  |
| 12-17 months | 2.87 | 2.57-3.21 | <0.001 | 3.16 | 2.81-3.56 | <0.001 |
| 18-23 months | 3.20 | 2.86-3.59 | <0.001 | 3.45 | 3.06-3.88 | <0.001 |
| Child’s sex |  |  |  |  |  |  |
| Male | Ref |  |  | Ref |  |  |
| Female | 1.09 | 0.99-1.19 | 0.073 | 1.05 | 0.96-1.16 | 0.300 |
| Maternal factors |  |  |  |  |  |  |
| Mother’s age |  |  |  |  |  |  |
| <24 years | Ref |  |  | Ref |  |  |
| 25-34 years | 1.22 | 1.09-1.36 | 0.001 | 1.00 | 0.89-1.13 | 0.945 |
| >35 years | 1.15 | 1.00-1.31 | 0.044 | 1.03 | 0.89-1.19 | 0.727 |
| Mother’s education |  |  |  |  |  |  |
| None or incompleted primary school | Ref |  |  | Ref |  |  |
| Completed primary school | 1.68 | 1.43-1.98 | <0.001 | 0.98 | 0.75-1.28 | 0.880 |
| Completed secondary school | 2.46 | 2.06-2.93 | <0.001 | 1.19 | 0.82-1.73 | 0.354 |
| Completed higher education | 3.87 | 3.16-4.75 | <0.001 | 2.96 | 1.67-5.25 | <0.001 |
| Mother’s occupation |  |  |  |  |  |  |
| Agricultural | Ref |  |  | Ref |  |  |
| Non-agricultural | 2.08 | 1.78-2.44 | <0.001 | 1.13 | 0.93-1.37 | 0.207 |
| Not working | 1.45 | 1.25-1.69 | <0.001 | 1.00 | 0.84-1.20 | 0.973 |
| Mother’s access to media at least once a week |  |  |  |  |  |  |
| None | Ref |  |  | Ref |  |  |
| Any media | 1.76 | 1.55-2.00 | <0.001 | 2.01 | 1.58-2.56 | <0.001 |
| Mother’s involvement in decision making |  |  |  |  |  |  |
| Not involved | Ref |  |  | Ref |  |  |
| Involved in any aspect | 1.31 | 1.08-1.59 | 0.005 | 1.19 | 0.97-1.47 | 0.089 |
| Paternal factors |  |  |  |  |  |  |
| Father’s education |  |  |  |  |  |  |
| None or incompleted primary school | Ref |  |  | Ref |  |  |
| Completed primary school | 1.31 | 1.12-1.55 | 0.001 | 0.94 | 0.78-1.14 | 0.508 |
| Completed secondary school | 1.82 | 1.54-2.14 | <0.001 | 0.98 | 0.79-1.20 | 0.822 |
| Completed higher education | 2.99 | 2.43-3.67 | <0.001 | 1.19 | 0.91-1.54 | 0.200 |
| Father’s occupation |  |  |  |  |  |  |
| Not working or agricultural | Ref |  |  | Ref |  |  |
| Non-agricultural | 1.74 | 1.58-1.93 | <0.001 | 1.23 | 1.09-1.40 | 0.001 |
| Health care, household, and community factors |  |  |  |  |  |  |
| Number of antenatal care visits |  |  |  |  |  |  |
| <4 | Ref |  |  | Ref |  |  |
| >4 | 1.81 | 1.57-2.07 | <0.001 | 1.21 | 1.04-1.40 | 0.014 |
| Postnatal care |  |  |  |  |  |  |
| No | Ref |  |  |  |  |  |
| Yes | 1.27 | 1.14-1.41 | <0.001 |  |  |  |
| Number of children under five within the household |  |  |  |  |  |  |
| <2 | Ref |  |  |  |  |  |
| >2 | 0.72 | 0.59-0.89 | 0.002 |  |  |  |
| Household wealth |  |  |  |  |  |  |
| Poorest | Ref |  |  | Ref |  |  |
| Poorer | 1.44 | 1.24-1.68 | <0.001 | 1.06 | 0.69-1.61 | 0.803 |
| Middle | 1.67 | 1.44-1.93 | <0.001 | 2.45 | 1.41-4.28 | 0.002 |
| Richer | 2.25 | 1.95-2.60 | <0.001 | 1.66 | 0.83-3.31 | 0.150 |
| Richest | 2.00 | 1.72-2.32 | <0.001 | 2.34 | 1.23-4.45 | 0.010 |
| Living residency |  |  |  |  |  |  |
| Urban | Ref |  |  | Ref |  |  |
| Rural | 0.95 | 0.94-0.96 | <0.001 | 0.97 | 0.96-0.99 | <0.001 |
| Region |  |  |  |  |  |  |
| Java and Bali | Ref |  |  | Ref |  |  |
| Sumatera | 0.95 | 0.85-1.06 | 0.365 | 1.09 | 0.97-1.23 | 0.149 |
| Kalimantan | 0.88 | 0.76-1.01 | 0.064 | 1.09 | 0.94-1.27 | 0.270 |
| Sulawesi | 0.66 | 0.58-0.75 | <0.001 | 0.89 | 0.69-0.91 | 0.001 |
| Eastern Indonesia | 0.51 | 0.44-0.58 | <0.001 | 0.71 | 0.61-0.84 | <0.001 |
| Household wealth # mother's education | | |  |  |  |  |
| Poorest # none or incompleted primary school | | | | Ref |  |  |
| Poorer # completed primary school | | |  | 1.94 | 1.27-2.96 | 0.002 |
| Poorer # completed secondary school | | |  | 1.45 | 0.88-2.43 | 0.146 |
| Poorer # completed higher education | | |  | 0.86 | 0.39-1.88 | 0.698 |
| Middle # completed primary school | | |  | 1.02 | 0.63-1.66 | 0.938 |
| Middle # completed secondary school | | |  | 1.03 | 0.59-1.80 | 0.927 |
| Middle # completed higher education | | |  | 0.55 | 0.26-1.14 | 0.109 |
| Richer # completed primary school | | |  | 2.70 | 1.42-5.12 | 0.002 |
| Richer # completed secondary school | | |  | 2.84 | 1.43-5.62 | 0.003 |
| Richer # completed higher education | | |  | 1.24 | 0.55-2.78 | 0.606 |
| Richest # completed primary school | | |  | 1.46 | 0.84-2.51 | 0.177 |
| Richest # completed secondary school | | |  | 1.64 | 0.89-3.03 | 0.112 |
| Richest # completed higher education | | |  | 0.96 | 0.46-2.03 | 0.920 |
| Household wealth # mother's access to media | | | |  |  |  |
| Poorest # none |  |  |  | Ref |  |  |
| Poorer # any media |  |  |  | 0.61 | 0.42-0.89 | 0.009 |
| Middle # any media |  |  |  | 0.45 | 0.29-0.69 | <0.001 |
| Richer # any media |  |  |  | 0.31 | 0.20-0.48 | <0.001 |
| Richest # any media |  |  |  | 0.40 | 0.24-0.67 | <0.001 |

OR: unadjusted odds ratios; AOR: adjusted odds ratios; Ref: reference; p: p-value

^a^Based upon the WHO indicator of minimum dietary diversity of at least 5 of 8 food groups in 2017 ^(13)^

^b^Independent variables adjusted for: survey year, child factors (age, sex), maternal factors (age, education, occupation, access to media, involvement in decision-making), paternal factors (education, occupation), and health care, household and community factors (ANC visits, postnatal care, household wealth, number of children, living residency, and region), interaction terms between household wealth and maternal education and household wealth and maternal access to media.
